# Embryonic Thermal Manipulation Affects Neurodevelopment and Induces Heat Tolerance in Layers

**DOI:** 10.3390/genes17010035

**Published:** 2025-12-30

**Authors:** Zixuan Fan, Yuchen Jie, Bowen Niu, Xinyu Wu, Xingying Chen, Junying Li, Li-Wa Shao

**Affiliations:** 1 Frontier Science Center for Molecular Design Breeding, State Key Laboratory of Animal Biotech Breeding, China Agricultural University, Beijing 100193, China; s20233040762@cau.edu.cn (Z.F.); s20203040572@cau.edu.cn (Y.J.); 2019304010115@cau.edu.cn (B.N.); s20243040788@cau.edu.cn (X.W.); s20253040866@cau.edu.cn (X.C.); lijunying@cau.edu.cn (J.L.); 2 Department of Animal Genetics and Breeding, National Engineering Laboratory for Animal Breeding, Key Laboratory of Animal Genetics, Breeding and Reproduction, Ministry of Agriculture and Rural Affairs, College of Animal Science and Technology, China Agricultural University, Beijing 100193, China

**Keywords:** embryonic thermal manipulation, layer, heat tolerance, neurodevelopment, molecular regulatory network

## Abstract

Background/Objectives: The poultry industry faces severe heat-stress challenges that threaten both economic sustainability and animal welfare. Embryonic thermal manipulation (ETM) has been proposed as a thermal programming strategy to enhance chick heat tolerance, yet its efficacy in layers requires verification, and its effects on growth performance and neurodevelopment remain unclear. Methods: White Leghorn embryos at embryonic days 13 to 18 (ED 13–18) were exposed to 39.5 °C (ETM). Hatch traits and thermotolerance were recorded, and morphometric and histopathological analyses were performed on brain sections. Transcriptome profiling of the whole brains and hypothalami was conducted to identify differentially expressed genes (DEGs). Representative pathway genes responsive to ETM were validated by RT-qPCR. Results: ETM reduced hatchability, increased deformity rate, and decreased hatch weight and daily weight gain. During a 37.5 °C challenge, ETM chicks exhibited delayed panting and lower cloacal temperature. Histopathology revealed impaired neuronal development and myelination. Transcriptomic analysis of ED18 whole brains showed DEGs enriched in neurodevelopment, stimulus response, and homeostasis pathways. RT-qPCR confirmed hypothalamic sensitivity to ETM: up-regulation of heat-shock gene *HSP70*, antioxidant gene *GPX1*, the inflammatory marker *IL-6*, and apoptotic genes *CASP3*, *CASP6*, *CASP9*; elevated neurodevelopmental marker *DCX*, indicative of a stress-responsive neuronal state; and reduced orexigenic neuropeptide *AGRP*. Conclusions: ETM improves heat tolerance in layers but compromises hatching performance and brain development, with widespread perturbation of hypothalamic stress responses and neurodevelopmental gene networks. These findings elucidate the mechanisms underlying ETM and provide a reference for enhancing thermotolerance in poultry.

## 1. Introduction

Poultry, a primary global source of animal protein, is integral to human diets and a key sector whose performance serves as an economic barometer for the livestock industry [1,2]. In recent years, intensified global warming has increased the frequency of extreme summer heat events, making heat stress a critical environmental challenge in poultry production [3,4]. Heat stress disrupts the dynamic balance between heat production and dissipation, causing heat to accumulate and precipitating physiological dysfunction [5]. Manifestations include hyperthermia, abnormal respiration, polydipsia, marked feed-intake reduction, growth retardation, depressed reproductive efficiency, and compromised immunity [6,7,8]. These changes directly impair poultry health and production efficiency, reduce economic returns and product quality, and challenge both animal welfare and the sustainability of the industry [2,9]. Notably, neonatal chicks—owing to their immature thermoregulation and high metabolic rate—are especially vulnerable to heat stress [10,11].

To mitigate the negative impacts of heat stress on poultry production performance, the industry has developed a multifaceted intervention system. Environmental regulation includes optimizing evaporative cooling systems to reduce barn temperatures [12]. Nutritional interventions include precise adjustments to dietary fat-to-amino acid ratios and supplementation of vitamin C [13,14]. Genetic improvement focuses on breeding heat-resistant strains [15]. In recent years, embryonic thermal manipulation (ETM)—a novel developmental programming technique—has emerged as a promising approach to enhance heat tolerance. This method applies thermal stress during critical embryonic developmental stages to improve post-hatch chick resilience to heat stress. For instance, Ncho et al. [16] demonstrated that broilers subjected to 39.6 °C on ED 10–18 exhibited lower rectal temperatures when challenged with 32 °C from post-hatched days (D) 29–35. Similarly, Al-Zghoul [17] found that broilers exposed to 38.5–40 °C on ED12–18 exhibited lower cloacal temperatures when challenged with 41 °C on D14 and D28. Piestun et al. [18] reported that broilers exposed to 39.5 °C on ED7–16 exhibited reduced corticosterone levels and mortality rates when challenged with 35 °C on D35. However, existing research remains fragmented, and studies on the efficacy of ETM in layers and newly hatched chicks are lacking. Thus, the generalizability of ETM for enhancing chicken heat tolerance across chicken breeds and developmental stages warrants further validation.

Furthermore, even if ETM enhances heat tolerance, its systemic effects on hatchability, chick quality, and subsequent production performance—particularly potential adverse effects—require comprehensive evaluation. Existing studies on ETM exhibit considerable variability in outcomes, likely attributable to differences in treatment parameters, including embryonic age, temperature, duration, and poultry breeds. Likewise, current research has focused predominantly on broilers, with limited investigations in layers. For instance, broiler embryos subjected to 38.5 °C during ED12–18 demonstrated improved hatchability and a shortened incubation period [19]. Conversely, broiler embryos exposed to 39.5 °C during ED7–16 exhibited reduced hatchability and lower hatchling body weight [18].

The molecular mechanisms underlying the brain’s response to ETM merit close scrutiny. Given its high metabolic rate, the brain is exquisitely thermosensitive [20]. The hypothalamus and associated regions serve as the central hub for sensing core body temperature and integrating peripheral thermal signals [21]. Through the coordinated regulation of endocrine, behavioral, and autonomic responses, these regions maintain thermal homeostasis [22]. Current evidence highlights the hypothalamus’s sophisticated regulatory machinery as indispensable for sensing and responding to heat stress. However, existing studies have predominantly focused on phenotypic observations in chickens, whereas systematic investigations into ETM’s impact on brain development and central regulatory networks remain scarce. This gap limits our comprehensive understanding of the neural mechanisms underlying heat stress regulation.

In this study, we subjected the layer egg-embryos to high-temperature incubation at 39.5 °C during the critical period of brain nerve maturation (ED13-18). Subsequently, we systematically evaluated core hatching traits, including hatchability, chick deformity rate, hatchling weight, daily weight gain, feed intake, and feed conversion ratio, while also detecting heat stress phenotypes such as panting and cloacal temperatures in young chicks under high-temperature conditions. Moreover, we collected whole brains and hypothalami from the embryos or chicks and employed a comprehensive approach integrating histopathology, transcriptome sequencing, and reverse transcription quantitative PCR (RT-qPCR) validation to systematically investigate neurodevelopment and underlying molecular regulatory networks. The purpose of this experiment was to elucidate how embryonic heat stress regulates heat tolerance, hatching performance, and neural development in layers, thereby providing a theoretical basis for developing heat tolerance enhancement strategies through early-life developmental programming.

## 2. Materials and Methods

### 2.1. Eggs and Incubation Management

Fresh fertile White Leghorn eggs were obtained from Boehringer Ingelheim Veyong Bio-tech Co., Ltd., Beijing, China. Eggs were stored under uniform conditions at 12–16 °C for <7 days and randomly allocated into two groups. 30 eggs per control group were incubated at a constant temperature of 37.8 °C and 65% relative humidity throughout the entire incubation period. 55 eggs per ETM group were exposed to 39.5 °C from ED13 to ED18; during all other periods, they were incubated under the same conditions as the controls. The number of eggs per group was based on previous studies [17,18]. Due to the lower hatchability of the ETM group, more eggs were incubated accordingly. Each egg was weighed and recorded before incubation. To minimize potential bias from vertical stratification or proximity to doors and ventilation openings, all eggs in each batch were placed in the same incubator, specifically on the middle shelf and in the inner zone. During the ETM treatment period, the eggs were transferred to an identical incubator of the same model and positioned equivalently to maintain consistency.

### 2.2. Hatchling Performance Evaluation

Relative egg weight (ED18/ED13) was calculated for each egg as the ratio of its weight on ED18 to its weight on ED13. Hatchability was determined as the number of chicks that hatched after 21 days of incubation divided by the total number of fertile eggs set (infertile eggs excluded). The deformity rate was calculated as the proportion of Day1 chicks exhibiting any of the following abnormalities: assisted hatching, unhealed abdomen, beak or limb defects, or sparse feathering. Each chick was weighed at approximately 12 h post-hatch, thereby minimizing the confounding effects of rapid post-hatch dehydration. Additionally, eight newly hatched chicks from each group were randomly selected, wing-tagged, and raised in individual cages. Feed consumption was recorded from Day1 to Day8. The average daily feed intake was calculated by dividing the total feed consumption by the number of chicks and days, and body weight was measured on Day8. The average daily gain during these 7 days was determined as the mean value obtained by subtracting the Day1 weight from the Day8 weight per chick and then dividing by days. The feed conversion ratio was calculated as the total feed consumption divided by the total body weight gain.

### 2.3. Heat Tolerance Test of Chicks

The brooding compartments, temperature-controlled and constructed from polystyrene foam boxes lined with aluminum-foam insulation panels, were each equipped with a heating lamp and a thermostat. Newly hatched chicks were randomly selected from each specific group and placed into these compartments. Temperature was maintained at 35.5 °C, 34.5 °C, and 33.5 °C on days 1, 2, and 3 post-hatch, respectively. Subsequently, eight chicks from each group were maintained at 37.5 °C. Over the following 7 days, the chicks’ panting behavior and cloacal temperatures were observed and recorded daily. On Day4, imaging was performed on chicks from each group.

### 2.4. Morphological and Pathological Analysis of the Brains

Day1 chicks were decapitated, and the whole brain—defined as the entire encephalic mass removed from the skull—was dissected and weighed. In addition, the term ‘brain’ used alone also denotes the whole brain. Relative brain weight was calculated as the ratio of brain weight to chick body weight, using five chicks per group.

Paraffin sections from brains were prepared for Hematoxylin and Eosin (HE), Toluidine Blue (TB), and Luxol fast blue (LFB) staining (three chick brains per group). Whole brains were fixed in 4% paraformaldehyde for 24 h, followed by dehydration in graded ethanol, clearing with xylene, and paraffin embedding. Sections (2–4 μm thick) were prepared using a rotary microtome and mounted on glass slides.

Histological staining (HE, TB, LFB, and Golgi staining) was performed by Wuhan Baqiandu Biotechnology Co., Ltd., Wuhan, China. For HE staining, sections were processed according to standard protocols: hematoxylin staining, eosin counterstaining, graded alcohol dehydration, and clearing. TB staining was performed with 0.1% TB solution, followed by differentiation in 1% glacial acetic acid under microscopic control. For LFB staining, sections were incubated overnight at 60 °C in 0.1% LFB solution. After rinsing, differentiation was performed using 70% ethanol and 0.05% lithium carbonate until the gray matter faded. Subsequently, sections were dehydrated, cleared, and mounted.

All mounted sections were scanned with a Pannoramic SCAN digital slide scanner (3DHISTECH Ltd., Budapest, Hungary), microscopic fields were randomly selected using SlideViewer v2.4, and cell analysis and statistics were performed using Image-Pro Plus 6.0 (Media Cybernetics, Rockville, MD, USA). For HE staining, ten 200× microscopic fields per section were evaluated. The vascular sheath, neurogliocytes, and disintegrating neurons were identified by their distinct coloration and morphology. For TB staining, six 200× microscopic fields per section were selected. Necrotic neurons were identified by deep nuclear blue staining and characteristic morphology. For LFB staining: Six 200× microscopic fields per section were analyzed. The percentage of myelinated area was calculated as the area of pixels whose blue-channel intensity exceeded a predefined threshold, divided by the total stained area.

For Golgi staining, three Day1 chick brains were stained in the dark at room temperature for 2–3 weeks. Frozen sections (100 µm thick) were prepared using a cryostat maintained at −20 to −22 °C, mounted on gelatin-coated slides and air-dried. After staining, destaining, and mounting, slides were scanned in bright-field mode (7-layer z-stacking). Six 400× microscopic fields per section were selected and evaluated. Dendritic spines within 30–90 µm segments were counted, and their density (spines per 10 µm) was calculated.

### 2.5. Transcriptome Sequencing and Analysis

Collected whole brains or hypothalami from ED18 chick embryos (three replicates per group). Tissues were homogenized in TRIzol (Invitrogen #15596018CN), and total RNA was extracted with chloroform, precipitated with isopropanol, and washed with 75% (*v*/*v*) ethanol. mRNA libraries were prepared and sequenced on the Illumina NovaSeq X Plus platform (2 × 150 bp paired-end) by Beijing Novogene Technology Co., Ltd., Beijing, China. FeatureCounts was used to count reads mapped to each gene. Differentially expressed genes between groups were identified with DESeq2 using a threshold of adjusted *p* < 0.05 and |log2 fold-change| > 1. Gene Ontology term enrichment analysis of biological processes was performed with DAVID Bioinformatics Resources 6.8 (National Institute of Allergy and Infectious Diseases, NIH, Bethesda, MD, USA).

### 2.6. RNA Isolation and Real-Time PCR

Whole brains or hypothalami from ED18 embryos or Day1 chicks were homogenized in TRIzol. Each sample comprised three biological replicates. Total RNA was extracted and cDNA was synthesized using the RT Reagent Kit (Takara, #RR047A). Quantitative PCR was performed with TB Green^®^ Premix Ex Taq ™ (Takara, #RR420A), and transcripts were normalized to GAPDH. Primer sequences are listed in Supplementary Table S1.

### 2.7. Statistical Analysis

Statistical analyses were conducted with GraphPad Prism 9.0 (GraphPad Software Inc., San Diego, CA, USA). Data are presented as mean ± SEM. *p*-values were calculated using two-tailed Student’s *t*-tests, following verification of normality and homogeneity of variance via Shapiro–Wilk and F tests, respectively, for all datasets. The significance levels for each figure are provided in the corresponding figure legends. A *p*-value < 0.05 was considered statistically significant. Except for RNA-seq, all other quantitative experiments were independently repeated at least three times, yielding consistent results.

## 3. Results

### 3.1. Embryonic Thermal Manipulation Affects Hatchling Performance and Heat Tolerance

**Figure 1:**
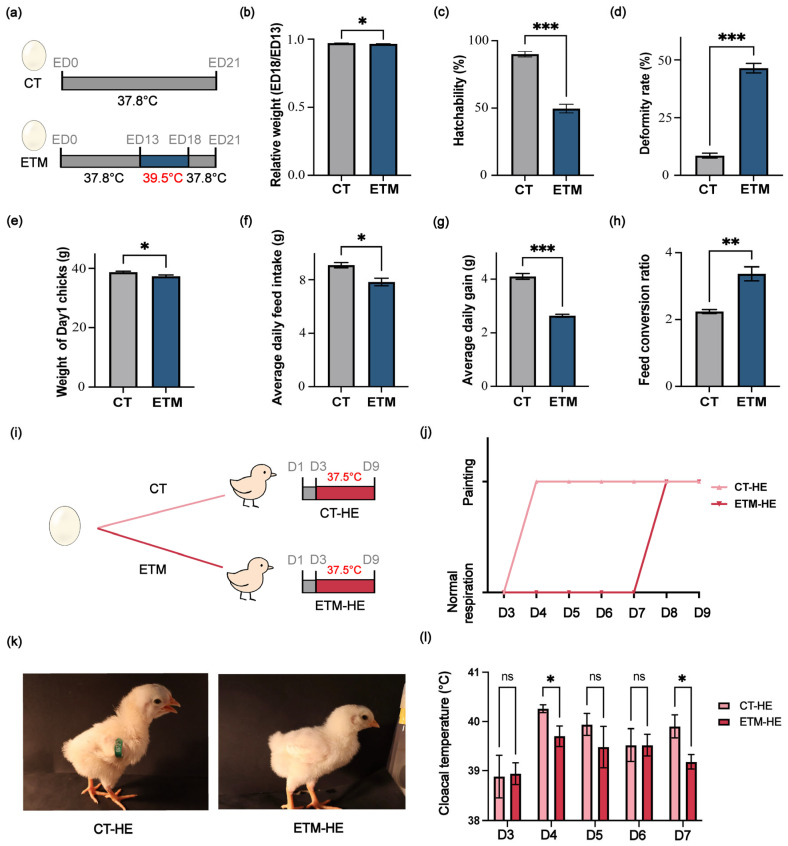
Effects of embryonic thermal manipulation on hatching chicks. (**a**) Schematic of the incubation conditions for the control (CT) and embryonic thermal manipulation (ETM) groups. Embryonic day, ED. ETM was performed at 39.5 °C from ED13 to ED18. (**b**) Relative weight of ED18 embryos versus ED13 embryos. (**c**) Hatchability of the indicated groups. (**d**) Deformity rate of the indicated groups. (**e**) Weight of the Day1 chicks in the indicated groups. (**f**–**h**) Average daily feed intake (**f**), daily gain (**g**) and feed conversion ratio (**h**) in chicks from days 1–8 post-hatch. (**i**) Schematic diagram of the treatment conditions for the two groups of newly hatched chicks. The post-hatched day, D; heat exposure, HE. (**j**) Temporal tracking of panting onset across indicated groups. (**k**) Panting phenotype of Day4 chicks in the indicated groups. (**l**) Cloacal temperatures of chicks in indicated groups. Data in (**b**–**h**,**l**) are presented as mean ± SEM. *p*-values were calculated by two-tailed Student’s *t*-test (* *p* < 0.05; ** *p* < 0.01; *** *p* < 0.001; ns, not significant).

Compared with the control (CT) group, which was incubated continuously at 37.8 °C for 21 days until hatch, the ETM group was subjected to heat treatment at 39.5 °C from ED13 to ED18 (Figure 1a). Initial egg weight at the onset of incubation was almost identical between the two groups (Figure S1). The relative egg weight (ED18/ED13) was calculated for both groups, confirming that the thermal manipulation results in increased egg weight loss (*p* < 0.05) (Figure 1b). The hatching rate in the ETM group significantly decreased to approximately 50% (*p* < 0.001) (Figure 1c), accompanied by a higher incidence of deformities (*p* < 0.001) (Figure 1d). Additionally, the body weight of newly hatched chicks was markedly reduced (*p* < 0.05) (Figure 1e). Post-hatch performance was then monitored for a week. Relative to CT chicks, ETM chicks showed significantly lower average daily feed intake (*p* < 0.05) (Figure 1f), reduced average daily gain (*p* < 0.001) (Figure 1g), and a higher feed conversion ratio (FCR) (*p* < 0.01) (Figure 1h).

Furthermore, heat tolerance in chicks was assessed by monitoring panting behavior and cloacal temperature. Chicks from the CT or ETM group were exposed to 37.5 °C from days 3 to 9 post-hatch (Figure 1i). CT-derived chicks (CT-HE) began panting on day 4, whereas ETM-derived chicks (ETM-HE) exhibited a delayed onset, initiating panting only on day 8 (Figure 1j,k). Additionally, following the heat challenge, their cloacal temperature was significantly lower than that of the CT group (*p* < 0.05) (Figure 1l).

### 3.2. Embryonic Thermal Manipulation Impedes Brain Development

**Figure 2:**
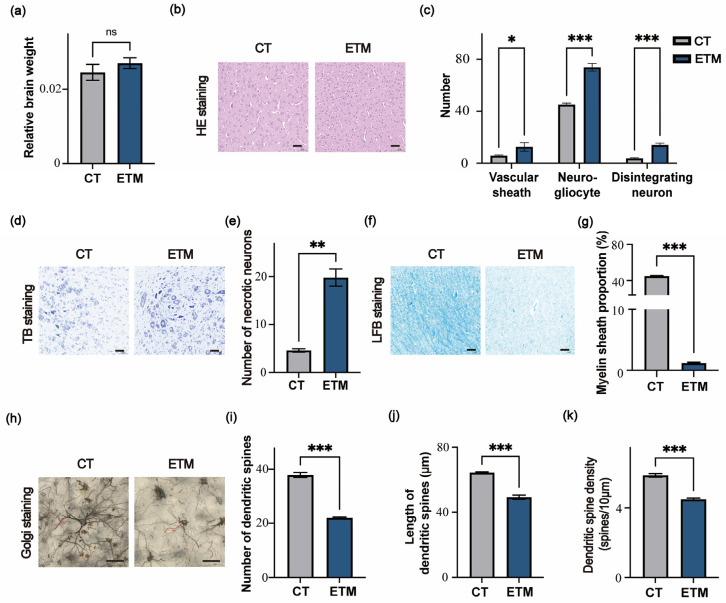
Effects of embryonic thermal manipulation on brain development. (**a**) Relative brain weight to body weight of the indicated Day1 chicks. (**b**,**c**) Hematoxylin and Eosin (HE) staining (**b**) of brain sections from the indicated Day1 chicks and quantification (**c**) of the vascular sheath, neurogliocytes, and disintegrating neurons. (**d**,**e**) Toluidine Blue (TB) staining (**d**) of brain sections from the indicated Day1 chicks and quantification (**e**) of the necrotic neurons. (**f**,**g**) Luxol Fast Blue (LFB) staining (**f**) of brain sections from the indicated Day1 chicks and quantification (**g**) of the myelin sheath proportion. (**h**–**k**) Golgi staining (**h**) of brain sections from the indicated Day 1 chicks, and quantification of dendritic spine number (**i**), dendritic spine length (**j**), and dendritic spine density (spines per 10 μm) (**k**). Red lines in (**h**) indicate randomly selected dendrites (30–90 µm). Scale bar, 50 μm. Data in (**a**,**c**,**e**,**g**,**i**–**k**) are presented as mean ± SEM. *p*-values were calculated by two-tailed Student’s *t*-test (* *p* < 0.05; ** *p* < 0.01; *** *p* < 0.001; ns, not significant).

The chicken brain coordinates systemic responses and serves as the central hub for life support and behavior. Whole brains from Day 1 chicks were isolated, weighed, and processed for either paraffin or frozen sections to assess neuropathological changes. Compared with the relative brain weight of the CT group, no significant difference was observed in the ETM group (*p* > 0.05) (Figure 2a). HE staining of paraffin sections revealed increased vascular sheaths, neurogliocytes, and nuclear-disintegrating neurons in ETM brains (*p* < 0.05) (Figure 2b,c). TB staining confirmed a marked rise in necrotic neurons in the ETM group (*p* < 0.01) (Figure 2d,e). LFB staining showed a pronounced reduction in myelinated area (*p* < 0.001) (Figure 2f,g). In addition, Golgi staining of frozen sections demonstrated a significant decrease in dendritic spine number, length and density (spines per 10 μm) in the ETM brains (*p* < 0.001) (Figure 2h–k).

### 3.3. Transcriptomic Profiling Uncovers Regulatory Networks in Brains Mediated by Embryonic Thermal Manipulation

**Figure 3:**
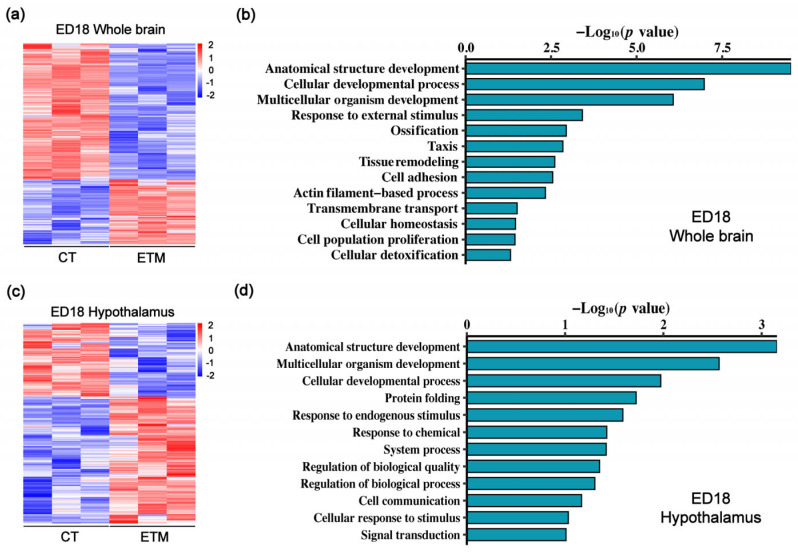
Transcriptomic responses of the whole brains and hypothalami to embryonic thermal manipulation. (**a**,**b**) Heatmap (**a**) and Gene Ontology (GO) analysis (**b**) of differentially expressed genes in ED18 whole brains transcriptomes from the indicated groups. (**c**,**d**) Heatmap (**c**) and GO analysis (**d**) of differentially expressed genes in ED18 hypothalamus transcriptomes. The heatmaps are row-scaled and colored by z-score: red indicates above-mean expression, blue below-mean. *p*-values in (**b**,**d**) were calculated by Fisher’s exact test and adjusted by the Benjamini–Hochberg method.

Transcriptome sequencing of whole brains from ED18 chicken embryos identified 993 differentially expressed genes (DEGs) using the selection criteria of *p* < 0.05 and |log2 fold-change| > 1 (Figure 3a). Gene Ontology (GO) enrichment analysis revealed that these DEGs were significantly associated with multiple biological processes, such as anatomical structure development, cellular developmental process, multicellular organism development, response to external stimulus, cell adhesion, transmembrane transport, cellular homeostasis, and cell population proliferation (Figure 3b).

As the central regulator of neuroendocrine function in chickens, the hypothalamus controls essential physiological processes such as thermoregulation, feeding behavior, and homeostasis. Transcriptome sequencing of hypothalamic tissue from ED18 embryos identified 333 DEGs (Figure 3c). These genes were significantly enriched in biological processes such as anatomical structure development, multicellular organism development, cellular developmental process, protein folding, response to stimulus, and regulation of biological quality (Figure 3d).

**Figure 4:**
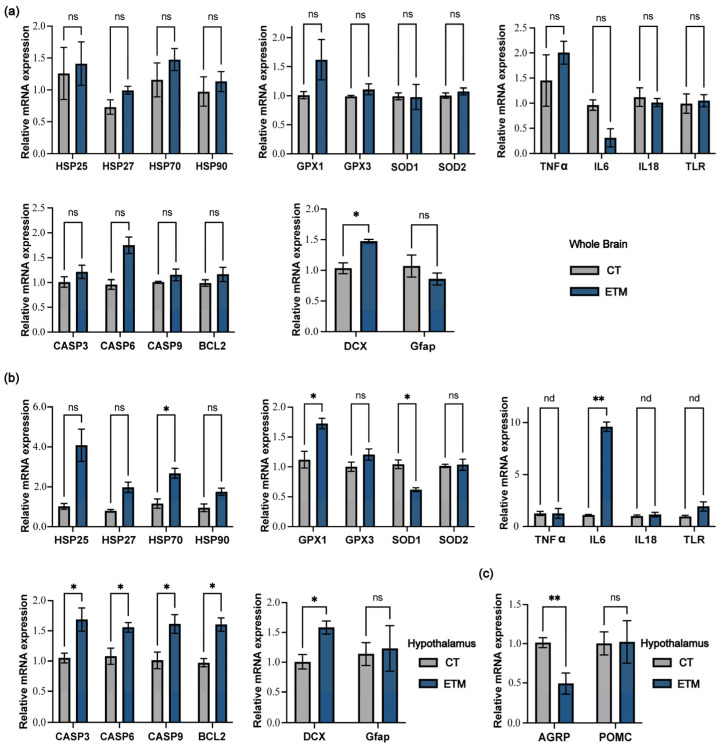
Responses of multiple key pathways in the whole brain and hypothalamus to embryonic thermal manipulation. (**a**) Reverse transcription quantitative PCR (RT-qPCR) analysis of mRNA expression for heat stress-related, oxidative stress-related, inflammation-related, apoptosis-related and neuron state-related genes in whole brains of ED18 embryos under the indicated conditions. (**b**) RT-qPCR analysis of the same genes as in (**a**) in the hypothalamus of ED18 embryos. (**c**) RT-qPCR analysis of appetite-regulating genes *AGRP* and *POMC* in the hypothalamus of Day1 chicks. Data are presented as mean ± SEM. *p*-values were calculated by two-tailed Student’s *t*-tests and adjusted for multiple comparisons via the Benjamini–Hochberg FDR correction (* *p* < 0.05; ** *p* < 0.01; ns, not significant).

To further clarify the response of chicken brains to ETM, we examined and compared the expression levels of multiple related genes in either the whole brains or the hypothalami of ED18 embryos from both groups. *DCX*, which governs neuronal migration and differentiation in the chicken brain and serves as a key marker of neurogenesis, showed significantly increased relative mRNA expression in the ETM group (*p* < 0.05) (Figure 4a). However, *DCX* is also expressed in immature or dysregulated neurons, and its up-regulation may reflect altered neurodevelopmental dynamics rather than solely functional neurogenesis. *GFAP*, a regulator of astrocyte activation and scar formation that reflects the state of glial response, did not change significantly (*p* > 0.05) (Figure 4a).

The hypothalamus showed a more pronounced response to ETM. The heat stress-related gene *HSP70*; oxidative stress-related gene *GPX1*; inflammation-related gene *IL6*; and apoptosis-related genes *CASP3*, *CASP6*, *CASP9*, and *BCL2* were all significantly upregulated in the ETM group (*p* < 0.05) (Figure 4b). *DCX* expression in the hypothalamus of the ETM group was also significantly increased (*p* < 0.05) (Figure 4b). Additionally, the hypothalamic gene *AGRP* promotes feeding behavior, whereas *POMC* suppresses appetite and regulates energy balance. RT-qPCR results from the hypothalamus of Day1 chicks showed that *AGRP* was significantly downregulated in the ETM group (*p* < 0.01) (Figure 4c).

## 4. Discussion

The layer industry is a strategically critical component of modern animal agriculture [23]. However, the convergence of genetically driven hyper-prolificacy, high-density husbandry, and global warming has positioned heat stress as a central bottleneck constraining production efficiency and animal welfare, while inflicting substantial annual economic losses on the global poultry sector [24,25]. Through systematic assessment, we found that ETM improves the heat resistance of White Leghorn chickens; however, it also significantly decreases hatchability, early body-weight gain, and normal brain maturation. Transcriptomic profiling further revealed that the hypothalamus exhibits genome-wide reprogramming of heat-shock, oxidative-stress, inflammatory, apoptotic, and neurogenic pathways.

Improved thermotolerance—manifested as delayed panting onset and lower cloacal temperature during heat stress—represents only one facet of ETM’s phenotypic outcomes. In terms of mechanism, embryonic heat stress may trigger a persistent molecular reprogramming in the hypothalamus, which can be viewed as a form of ‘stress imprinting’. This reprogramming establishes a state of heightened alertness to subsequent heat challenges, characterized by the pre-activation of cytoprotective pathways. The HSP protein HSP70 is markedly up-regulated, which not only safeguards protein conformational stability [26,27], but also restrains IKK–NF-κB-driven hyper-inflammation via the HSF1–HSP axis, thereby preserving the stability of the thermoregulatory set point [28]. Activation of *GPX1* may reinforce the antioxidant system in advance, attenuating ROS-mediated secondary neuronal injury [29,30]. Enhanced neuromodulatory capacity—mediated by neurotransmitters and hormonal signals—enables neonatal chicks to rapidly re-establish thermal and metabolic homeostasis after hatch [20]. Pre-activation of the immune–antioxidant machinery further elevates the organismal threshold for heat-induced inflammation [31]. However, it is crucial to note that this imprinted state is not benign; it is established concurrently with evident developmental disruption, as evidenced by our histopathological findings. Thus, the upregulation of these pathways likely represents a double-edged sword: an attempt to orchestrate cellular defense and survival in the face of thermal insult, yet occurring within a context of measurable tissue injury and compromised neurodevelopment—highlighting the need for further functional validation.

The impact of embryonic heat exposure on brain development has become a focal research theme. In the present study, relative brain weight was slightly—albeit not significantly—higher in the heat-manipulated chicks than in controls, indicating that gross brain mass alone is insufficient to capture functional injury induced by thermal stress. Subsequent histological analyses revealed that the ETM group exhibited markedly increased vascular sheaths, gliocyte and neuronal necrosis, together constituting a canonical stress-related inflammatory profile. An increase in vascular sheaths may indicate increased intramural inflammatory-cell infiltration and impaired vascular-barrier function [32], whereas glial activation and proliferation—through phagocytosis of dying neurons and release of anti-inflammatory mediators—represent a compensatory attempt to restore local homeostasis [33]. Yet this compensatory response is insufficient to fully counteract thermal damage, as evidenced by a significant reduction in myelinated area, suggesting impaired oligodendrocyte differentiation or compromised axonal integrity, and consequently, decreased efficiency of neural signal transmission. Concomitant decreases in dendritic spine number, length, and density further indicate suppressed synaptogenesis and diminished synaptic plasticity, plausibly attributable to diminished neurotrophic support and impaired synthesis of synaptic proteins [34]. Transcriptomic data corroborate these morphological findings: up-regulation of the inflammatory marker *IL-6*, together with concurrent elevations in pro-apoptotic (*CASP3/6/9*) and anti-apoptotic (*BCL2*) transcripts, imply that thermal stress simultaneously triggers immune-inflammatory cascades and apoptotic programs [35], whereas elevated *DCX* expression suggests altered neurodevelopmental dynamics that may contribute to network remodeling, reinforcing the notion that the ETM-induced molecular reprogramming encompasses both protective and detrimental elements. Collectively, embryonic thermal manipulation exerts deleterious effects on neuronal survival, myelination, and synaptic plasticity, and the functional relevance and molecular underpinnings of this process merit in-depth investigation. Besides, cell-type-specific analyses in future work may help delineate more precise mechanisms.

Embryonic development is extremely sensitive to temperature fluctuations [36,37]. Hyperthermia influences embryonic development by impairing energy metabolism, limiting nutrient utilization, suppressing organogenesis, exacerbating oxidative stress, and inducing aberrant epigenetic remodeling [19,38,39,40,41]. Heat increases oxygen consumption and energy demand; if the stressor is sufficiently intense, vitelline angiogenesis is blunted and yolk-sac resorption is delayed, diminishing the efficiency of nutrient and gaseous exchange and ultimately manifesting as dual reductions in body mass and hatchability [19,38]. Growth of the heart, liver, and immune organs is suppressed, accompanied by elevated heart rate, increased CO_2_ emissions, and compromised immune competence [19,39]. Excessive reactive oxygen species (ROS) disturb redox homeostasis and further trigger cellular damage and apoptosis [40,42]. In addition, altered DNA methylation landscapes and imbalanced expression of immune-related genes weaken developmental potential [41].

Within this mechanistic framework, the present study provides some empirical evidence. First, hypothalamic *AGRP* expression was significantly down-regulated, corroborating the report by He et al. [43] that heat suppresses appetite-related gene expression, and diminished feeding motivation may contribute to inadequate nutrient intake. Second, sustained up-regulation of heat-shock proteins assists in maintaining proteostasis but incurs additional energetic costs and can inhibit protein synthesis via the AMPK–mTOR axis, thereby amplifying the imbalance in energy allocation [44]. Thus, the ETM-induced decline in body mass is not solely attributable to appetite suppression but also reflects a systemic trade-off in which metabolic resources are re-allocated toward thermotolerance at the expense of growth. Future studies that integrate dynamic sampling, multi-tissue profiling, and single-cell resolution are expected to clarify the temporal hierarchy and tissue specificity of these effects, thereby providing a comprehensive dissection of the molecular trade-offs underlying enhanced thermotolerance and growth restriction.

The impact of ETM on broiler performance is highly contingent upon the treatment condition. Although the ETM condition used here is partially similar to previously reported settings [38,45], it differs in the exact temperature and incubation period used. Earlier studies in broilers showed that a moderate temperature increase (38.5–40 °C) during mid or late incubation either has no effect on or only slightly reduces hatchability [18]. In contrast, when the temperature exceeds the embryonic metabolic threshold (~40 °C) or the treatment is prolonged, development is impaired, hatchability drops and mortality rises [46]. More specifically, exposing embryos to 39.0 °C from ED10–18 did not influence hatchability [47], whereas 39.6 °C during the same period reduced it [48]. Likewise, heat exposure at 40.6 °C during ED16–18 restricted embryonic growth, decreased yolk utilization, delayed chick hatching, and markedly lowered hatchability by increasing embryonic mortality, while simultaneously disturbing carbohydrate and lipid metabolism [49]. On the other hand, ETM can enhance subsequent thermotolerance, as evidenced by improved thermoregulatory capacity, a lower respiratory rate, and favorable endocrine and immune modulation [39,50]. When a daily 39.5 °C was applied during the critical ED7–16 window for hypothalamic–pituitary–thyroid axis development, broiler chicks hatched with a persistently lower body temperature, higher venous blood oxygen saturation and lower partial pressure of CO_2_ under heat challenge; the heterophil-to-lymphocyte ratio did not increase significantly, indicating improved physiological homeostasis and thermotolerance without compromising hatchability [51]. Similarly, Loyau et al. [50] discontinuously exposed embryos to 39.5 °C from ED 7–16 and subsequently reported a delayed rise in body temperature and reduced heart rate in ETM chicks during heat exposure. Al-Zghoul et al. [39], who applied 39 °C from ED 10–18, observed up-regulated immune-related gene expression and higher antibody levels.

In the present study, continuous 39.5 °C treatment from ED13–18 improved post-hatch thermotolerance of laying hens, yet it simultaneously reduced hatchability, hatch weight and daily weight gain, and induced brain morphology abnormalities. It should be noted that all post-hatch measurements could only be derived from a subset of surviving embryos, introducing a potential survivorship bias, particularly in the ETM group. In addition, the absence of an a priori power analysis suggests that the present sample may have been underpowered. Moreover, the absence of sex information is a limitation of this study. Although randomization was used to minimize any sex-related effects, we cannot completely rule out the potential influence of sex on growth and development. The feed intake and FCR data presented in this study were collected only during the first eight days post-hatch. This period corresponds to a critical transitional phase during which chicks adapt from embryonic yolk-based nutrition to exogenous feeding. The observed reduction in feed intake and impaired FCR in ETM chicks coincided with a significant downregulation of hypothalamic *AGRP*. This suggests that ETM may modulate central appetite-regulatory circuits, potentially altering the metabolic set point and early feeding behavior. These findings primarily reflect the impact of ETM on metabolic adaptation and the establishment of feeding patterns during the early post-hatch stage. Nevertheless, the early reductions in feed efficiency and body weight gain may signal a potential risk for long-term production performance.

Taken together, our data paint a picture of physiological trade-offs induced by ETM. On one hand, the pretreatment with moderate heat stress during a critical window of embryogenesis appears to ‘prime’ the hypothalamic stress-response machinery. The coordinated upregulation of HSPs, antioxidant enzymes, and anti-apoptotic factors may contribute to the observed improved thermotolerance in chicks, as indicated by delayed panting and attenuated hyperthermia during heat challenge. On the other hand, this priming comes at a substantial cost. The same treatment severely compromises hatchability, early growth performance, and normal brain maturation. The histopathological evidence of neuroinflammation, neuronal necrosis, impaired myelination, and reduced synaptogenesis, coupled with the transcriptional signatures of inflammation and apoptosis, unequivocally demonstrates that the embryonic brain experiences significant stress and damage under our ETM protocol. Therefore, the ‘heat-tolerant’ phenotype emerging from ETM should be interpreted not as an unalloyed benefit, but as the outcome of a developmental prioritization where resources are diverted towards survival and stress resilience at the expense of optimal growth and neural integrity.

The approximately 50% reduction in hatchability and the clear evidence of developmental impairments observed in this study constitute substantial practical and ethical barriers to the commercial application of this ETM protocol. To further enhance heat tolerance through embryonic thermal programming, future studies should prioritize optimizing the specific parameters of heat exposure to ensure programming efficacy while minimizing physiological disruption. Concurrently, the incorporation of antioxidants—such as N-acetylcysteine or vitamin E—during incubation should be explored to mitigate oxidative damage and improve hatchability. Moreover, the experimental scale—such as the number of eggs and RNA-seq replicates—should be expanded on the basis of preregistered power calculations, sex should be determined, and long-term follow-up studies should be conducted to systematically assess the effects of ETM on brain development, egg-laying performance, behavior, and overall welfare in laying hens.

## 5. Conclusions

Our study provides a comprehensive evaluation of ETM as a strategy to enhance heat tolerance in laying hens. While ETM at 39.5 °C from ED13–18 improved thermotolerance post-hatch—evidenced by delayed panting and reduced cloacal temperature under heat stress—it concurrently imposed significant trade-offs. These included reduced hatchability, increased deformity rates, impaired early growth performance, and marked disruptions in brain development. Histopathological and transcriptomic analyses revealed widespread neural damage and molecular reprogramming, particularly within hypothalamic stress-response, neurodevelopmental, and metabolic pathways. These findings underscore the dual nature of ETM: it offers potential adaptive benefits while incurring developmental costs. Future work should focus on optimizing thermal protocols and integrating supportive interventions to maximize thermotolerance gains while minimizing adverse effects on hatchability and neurodevelopment.

## Data Availability

The datasets generated and analyzed during the current study are available in the GEO repository (GSE306311) at https://www.ncbi.nlm.nih.gov/geo, accessed on 1 January 2026.

## Supplementary Materials

The following supporting information can be downloaded at: https://www.mdpi.com/article/10.3390/genes17010035/s1.
