# Supplementary material for: Embryonic Thermal Manipulation Affects Neurodevelopment and Induces Heat Tolerance in Layers
Source: Genes (Basel). 2025 Dec 30;17(1):35. doi: 10.3390/genes17010035 (PMC12840955; doi:10.3390/genes17010035)
Supplement: Supplementary file 1 [file genes-17-00035-s001.zip › supplementary materials/Supplementary Figure 1.pdf]

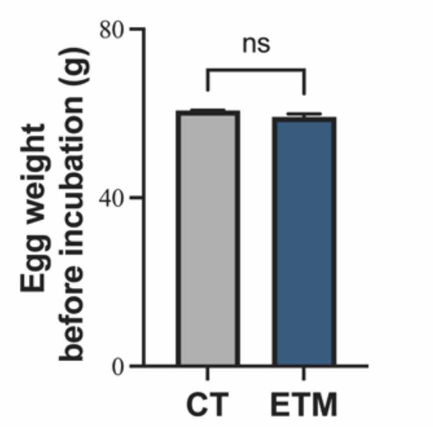

Fig. S1 Egg weight before incubation in the control (CT) and embryonic thermal manipulation (ETM) groups. Data are presented as mean ± SEM. *p*-value was calculated by two-tailed Student's t test (ns, not significant).
