# Supplementary material for: Embryonic Thermal Manipulation Affects Neurodevelopment and Induces Heat Tolerance in Layers
Source: Genes (Basel). 2025 Dec 30;17(1):35. doi: 10.3390/genes17010035 (PMC12840955; doi:10.3390/genes17010035)
Supplement: Supplementary file 1 [file genes-17-00035-s001.zip › supplementary materials/Supplementary Table 1.pdf]

Supplementary Table 1. Primer sequences used for RT-qPCR

Gene	Primer Sequence (5’→3’)
HSP25	F:AGAGACCATCTTCAGCGAGC<br>R:TTCTTCACATCCTGGCAGACG
HSP27	F:CGGCAAACACGAGGAGAA<br>R:GGCCTCCACTGTCAGCATC
HSP70	F:GCGCCAGGCCACCAAAGATG<br>R:GCCCCCTCCCAAGTCAAAGATG
HSP90	F:AAGGCAAGACCCTGGTTTCT<br>R:CCAAGCGATTGGACACAACA
GPX1	F:CAATTCGGGCACCAGGAGAA<br>R:GTACTGCGGGTTGGTCATCA
GPX3	F:GGGCACCATCTACGACTACG<br>R:TGTAGTGCATTCAGTTCGAGG
SOD1	F:CCAAAAGATGCAGATAGGCACG<br>R:GCAGTGTGGTCCGGTAAGAG
SOD2	F:GTGGAGGAGGGGAGCCTAAA<br>R:CAGCAATGGAATGAGACCTGTTG
TNFα	F:GGGACATCTCTCCAGGGGAA<br>R:CGAGCACTGCATTGTGTCT
IL6	F:CGATCCGGCAGATGGTGATA<br>R:CAGAGGATTGTGCCCGAACT
IL18	F:GAAGAGATCGCTGTGTGTGC<br>R:ATCGCATTCCAGCTCATCAT
TLR	F:TGGATCTTTCAAGGTGCCACA<br>R:AGTGTCCGATGGGTAGGTCA
CASP3	F:TGGTGGAGGTGGAGGAGC<br>R:CATCTTCCCCTGAGCGTGG
CASP6	F:ATGTCGGGCGCGGAGCGGCGGCCGG<br>R:CCGGCCGCCGCTCCGCGCCCGACAT
CASP9	F:AACCTTGGACAGCGTACTGG<br>R:CGATGTCTGACACCCGAAGT
BCL2	F:GGATCGTCGCCTTCTTCGAG<br>R:CCACAAAGGCATCCCATCCTC
DCX	F:GCAGCTGCCACAGGTAGTAA<br>R:CTCGAGGTCCCATTTGCTGA
GFAP	F:ACCAGCCTGGACACCAAATC<br>R:TGGACTCCTTGATCACCTCT
AGRP	F:GGTGCAGGAAGGTGATGGTAA<br>R:GCGTTGAAGAACCGGCAGTA

POMC	F: CATGCTGGGAGAACAGCAAGT<br>R: GACGGCCGAACTTGTTCCAG
GAPDH	F: ACTATCTTCCAGGAGCGTGAC<br>R:ACCCATCACAAACATGGGGG
